# Modelling the potential health and economic benefits of reducing population sitting time in Australia

**DOI:** 10.1186/s12966-022-01276-2

**Published:** 2022-03-19

**Authors:** Phuong Nguyen, Jaithri Ananthapavan, Eng Joo Tan, Paul Crosland, Steve J. Bowe, Lan Gao, David W. Dunstan, Marj Moodie

**Affiliations:** 1 Deakin Health Economics, Deakin University, Institute for Health Transformation, Geelong, VIC Australia; 2 Global Obesity Centre, Deakin University, Institute for Health Transformation, Geelong, VIC Australia; 3 Biostatistics Unit, Faculty of Health, Deakin University, Geelong, VIC Australia; 4 School of Biomedical Sciences and Pharmacy, The University of Newcastle, Callaghan, NSW Australia; 5 Baker Heart and Diabetes Institute, Melbourne, Australia; 6 Mary MacKillop Institute for Health Research, Australian Catholic University, Melbourne, VIC Australia

**Keywords:** Sedentary behaviour, Health benefits, Healthcare cost, Modelling, Cost-of-illness

## Abstract

**Background:**

Strong evidence indicates that excessive time spent sitting (sedentary behaviour) is detrimentally associated with multiple chronic diseases. Sedentary behaviour is prevalent among adults in Australia and has increased during the COVID-19 pandemic. Estimating the potential health benefits and healthcare cost saving associated with reductions in population sitting time could be useful for the development of public health initiatives.

**Methods:**

A sedentary behaviour model was developed and incorporated into an existing proportional, multi-state, life table Markov model (ACE-Obesity Policy model). This model simulates the 2019 Australian population (age 18 years and above) and estimates the incidence, prevalence and mortality of five diseases associated with sedentary behaviour (type 2 diabetes, stroke, endometrial, breast and colorectal cancer). Key model inputs included population sitting time estimates from the Australian National Health Survey 2014–2015, healthcare cost data from the Australian Institute of Health and Welfare (2015) and relative risk estimates assessed by conducting literature reviews and meta-analyses. Scenario analyses estimated the potential change in disease incidence as a result of changes in population sitting time. This, in turn, resulted in estimated improvements in long term health outcomes (Health-adjusted life years (HALYs)) and healthcare cost-savings.

**Results:**

According to the model, if all Australian adults sat no more than 4 h per day, the total HALYs gained would be approximately 17,211 with health care cost savings of approximately A$185 million over one year. Under a more feasible scenario, where sitting time was reduced in adults who sit 4 or more hours per day by approximately 36 min per person per day (based on the results of the Stand Up Victoria randomised controlled trial), potential HALYs gained were estimated to be 3,670 and healthcare cost saving could reach A$39 million over one year.

**Conclusions:**

Excessive sedentary time results in considerable population health burden in Australia. This paper describes the development of the first Australian sedentary behaviour model that can be used to predict the long term consequences of interventions targeted at reducing sedentary behaviour through reductions in sitting time. These estimates may be used by decision makers when prioritising healthcare resources and investing in preventative public health initiatives.

**Supplementary Information:**

The online version contains supplementary material available at 10.1186/s12966-022-01276-2.

## Introduction

The association between sedentary behaviour (SB) and chronic diseases has been established, with strong evidence of associations between high sedentary time and the risk of type two diabetes (T2D) [1–5] and cardiovascular disease (CVD) [1–3, 5, 6], and moderate evidence of its association with various cancers [1–3]. In acknowledgement of its distinction from physical inactivity (not meeting physical activity recommendations), SB has now been incorporated into public health guidelines, and refers to any waking behaviour characterised by low energy expenditure of less than 1.5 metabolic equivalent of tasks (METs) [7], while in a sitting or lying position. Light intensity PA, on the other hand, ranges between 1.6—3 METs, e.g. slow walking or standing with minor effort; and moderate intensity PA ranges between 3.0 – 6.0 METs (e.g. brisk walking, light cycling) [8]. A person could meet physical activity recommendations by doing at least 30 min of moderate activity on most days but may still be sedentary by accumulating more than 8 h of sitting (including sitting at work, during transportation or leisure time). Frequently, the terms ‘sitting time’ or ‘sedentary time’ have been used interchangeably with SB.

The prevalence of SB is high in Australia. The Australian National Health Survey (NHS) for Physical Activity 2011–12 reported that adults on average spent 39 h per week (5.7 h per day) in SB across the domains of work, leisure and transport [9]. The most recent Australian NHS 2017–18 reported that approximately 44% of adults aged 18–64 years describe their work day as mostly involving sitting [10].

Interventions and policies to address the high prevalence of SB could contribute to the reduction of the burden of various chronic diseases. Over the past decade, there has been an increased focus on interventions to reduce sitting time. Although early studies have shown mixed results [11], more recent systematic reviews have consistently shown that interventions can be effective, with meta-analyses reporting a statistically significant effect size ranging from 40 to 100 min per 8-h workday for workplace interventions [12–14]. Cost-effective analyses have been conducted for some SB interventions [15–18]. However, prior health economic modelling has typically focused on translating changes in population-level physical activity, rather than capturing the reduction in SB. Awareness of the economic burden associated with SB could inform and motivate policymakers to address this risk factor. Specifically, estimates of the potential health benefits and healthcare cost savings that could be achieved from reductions in population sitting could provide decision makers with the economic arguments to prioritise funding and investment in prevention. While the evidence on the burden arising from other modifiable risk factors such as poor diets, physical inactivity, smoking, and alcohol consumption is well established in Australia and worldwide [19], there is limited evidence on the health and economic burden of SB. To our knowledge, only one study in the UK has estimated the direct healthcare costs associated with SB [20]. Heron et al. 2019 [20] reported that excessive sitting would cost the UK healthcare system the substantial sum of GBP761.80 million annually.

The aim of the current study was to estimate the potential health benefits and healthcare cost savings arising from potential reductions in excessive sitting in the Australian population. This study involved developing a SB model that would be integrated as a new risk factor into an existing model, the ACE-Obesity Policy model [21, 22]. This paper outlines the steps in developing this model and reports the results of scenario analyses that estimate the potential benefits and savings associated with reductions in SB.

## Methods

### Overview of the modelling

The ACE-Obesity Policy model is a proportional, multi-state, life table Markov model simulating the body max index (BMI), physical activity (PA) and fruit and vegetable consumption profile of the 2010 Australian population [22]. The model calculates the health adjusted life years (HALYs) saved as a result of an intervention’s effectiveness in improving any of the above risk factors for obesity, captured through reductions in prevalence of nine obesity-related diseases: breast cancer, endometrial cancer, kidney cancer, hypertensive heart disease, ischemic heart disease, stroke, T2D and osteoarthritis (hip and knee). The ACE-Obesity Policy model has been used in economic evaluations of multiple obesity prevention interventions [23–27], including two SB reduction interventions [22]. However, both these evaluations used the PA component of the model and estimated the changes in physical activity (resulting from increased standing measured using METs) arising from the SB intervention. One intervention in children assumed the resultant reduction in sitting time was equal to an increase in standing time [21]; the other workplace intervention modelled an increase in standing time [16]. The direct impact of SB on chronic disease was not estimated. Given that SB is not equivalent to physical inactivity, this current research sets out to more accurately estimate the impact of reductions in SB using epidemiological and economic modelling. The SB module allows the direct modelling of intervention outcomes through reductions in sitting time rather than increases in physical activity. The SB model will be integrated into the current ACE-Obesity Policy model to facilitate the comparative analysis of interventions with impacts on various risk factors.

The steps involved in developing and incorporating the SB risk factor model into the ACE-Obesity Policy model include: (1) assessment of the current Australian adult population exposure to sitting time; (2) a systematic review of the current literature to identify the associations between SB and incidence of chronic diseases, in particular the nine diseases included in the ACE-Obesity Policy model, and the conduct of a meta-analyses; and (3) translation of the reduction in population sitting time into decreases in disease incidence using potential impact fractions (PIF). The primary parameters of the existing ACE-Obesity Policy model such as population, all-cause mortality, disease inputs (incidence, prevalence and case fatality) and disease costs were updated from 2010 to 2019 values using various sources.

### Assessing population sitting time

In order to establish the risk of disease associated with SB, distinct levels of SB needed to be defined. In this analysis, three distinct categories of self-reported SB were used, namely low SB (< 4 h of sitting time per day), moderate SB (4–8 h), and high SB (> 8 h of sitting time per day). These categories are consistent with those used by Van Der Ploeg and colleagues [28] to establish mortality risk associated with SB in a cohort of Australian adults. Further, Clark et al. 2015 [29] reported a discrepancy between self-reported and device-measured sitting time (activPal); self-reported measurements were under-reported by 2 h. Thus, applying the 8 h cut-off for the self-reported measure as the upper limit brings this in line with Ekelund et al. 2019 which showed that 9.5 h of sedentary behaviour (device measured) is the level at which risk is substantially elevated [30].

Weighted mean sitting times by age and gender groups for the three SB categories were based on data extracted from The Australian National Health Survey 2014 basic CURF (Confidentialised Unit Record Files) [31]. The analysis was conducted using Stata and jackknife estimator for survey analysis. These mean total sitting times included both workplace and leisure sitting time.

### Assessing the association between sitting time and chronic diseases

The association between SB and chronic diseases of interest for this model was estimated by first undertaking a review of the published literature. The literature has been comprehensively synthesised in the US Physical Activity Guidelines Advisory Committee 2018 report [1] and was updated in 2019 [2]. However, it is noted that this level of evidence was based on the quantitative analyses of all types of CVD combined, and all cancers combined; to accurately estimate the costs and health benefits of interventions, disease specific relative risks (RR) were required. Cohort studies from these two comprehensive systematic reviews [1, 2] were selected to investigate the association between sitting time and specific chronic diseases. The review papers from these reports were hand searched to identify additional relevant cohort studies. Data on study design, participants, and the association (RR or hazard ratio (HR)) between SB and disease incidence were extracted. The RR was chosen from the multi-variants regression models which included adjustment for PA level. RR and HR were considered to be interchangeable (see Appendix 1 for further discussion). Separate meta-analyses were conducted for each outcome (breast cancer, colorectal cancer, endometrial cancer and T2D) using random-effect model with the restricted maximum likelihood method (REML). Heterogeneity was assessed using the I^2^ statistic. Results were reported as overall relative risk and 95% confidence intervals (CI). Further details of the inclusion and exclusion criteria, methodology used and meta-analysis results, including subgroup analyses of high and moderate sedentary behaviour, are reported in Appendix 1.

### Sedentary behaviour model

**Fig. 1:**
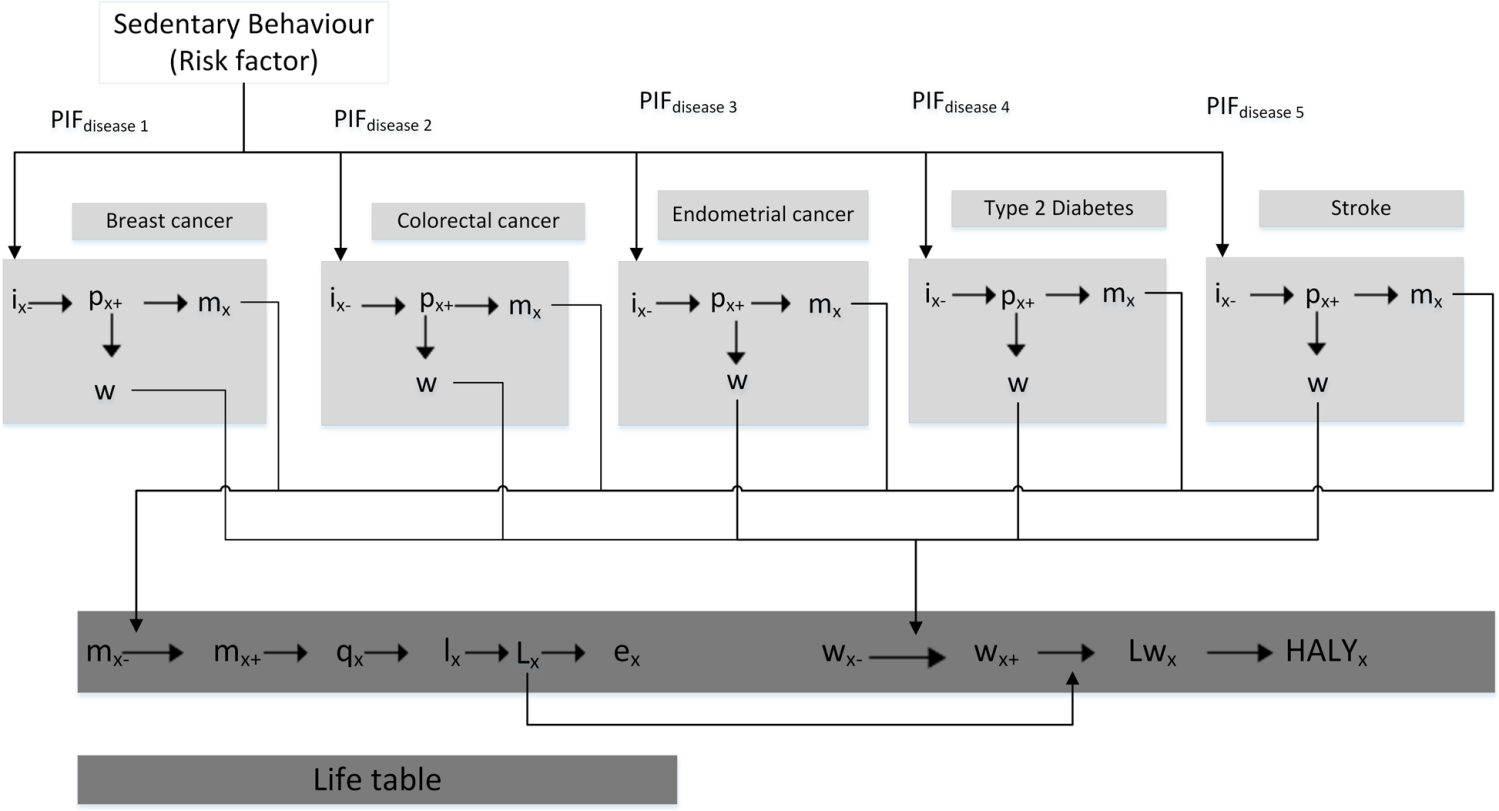
Proportional multi-state life table (Adapted from Forster et al. [34]; PIF: potential impact fraction; x: age; i: incidence; p: prevalence; m: mortality; w: disability-adjustment; q: probability of dying; l: number of survivors; L: life years; Lw: disability-adjusted life years; HALY is health-adjusted life year; -: denotes a parameter that is related to all other diseases but excludes modelled diseases; + : denotes a parameter that relates to all modelled diseases). This schematic demonstrates how HALYs were calculated from the disease process of five diseases

The SB model calculates the difference in the epidemiology (incidence, prevalence, and mortality) of the SB-related diseases for a population exposed to intervention resulting in changes in sitting time and an identical population without the intervention, i.e. no changes in the SB profile (status quo of the current population). Figure 1 shows a schematic of the multi-state life table model. Within this theoretical framework, the changes in sitting time lead to reduced incidence of diseases, which then leads to reduced prevalence and disease-related mortality and morbidity. These, in turn, result in improved long term health outcomes (HALYs) and healthcare cost-savings. HALY is an umbrella term for health related QoL measures allowing morbidity and mortality to be simultaneously cooperated within a single number [32]. In the ACE studies, HALYs are calculated in the same way as a QALYs (remaining years of life are weighted with quality of life), however rather than using utility weights, disability weights from the GBD are used [33].

For each of the diseases included in this model, PIFs were used to calculate the proportional change in disease incidence (Fig. 1). The PIF is a measure of the change in incidence of a disease as a result of changed exposure to a risk factor [35]. PIFs were derived from three key parameters: the population prevalence and mean sitting time associated with SB categories before and after the intervention and the relative risk of SB-related diseases. PAFs have been used in previous studies estimating the burden of disease associated with SB [20], however the PIF is a more appropriate estimate as the counterfactual for the unexposed is not zero sitting time [36]. The use of PIFs is, therefore, more appropriate for evaluating population interventions where the exposure to a risk factor is a continuous phenomenon. Compared to PAFs, PIFs are mostly calculated using a categorical risk factor distribution and a relative risk for each category [35].

Given that the RR of disease is based on SB categories, the relative-risk shift method was used to calculate PIFs as this methods has been reported to be a simple and suitable for evaluating population interventions addressing categorical risk factors [35]. RRs were modelled using a normal distribution, and sitting time was modelled using a lognormal distribution. This approach was adopted from the method used in the PA risk factor module in the ACE Obesity Policy model [22, 37].

Disease-specific life tables were used to calculate the overall epidemiologic impact of a reduction in sitting time on SB-related diseases. A generic disease model described in Barendregt et al. 2003 [38] was used to calculate the epidemiologic impact of reductions in sitting time on each of the included diseases. Each disease consisted of four health states namely “healthy”, “diseased”, “dead due to the disease” and “dead due to other causes” (Appendix 2). DisMod II software was used to calculate age and sex specific transition probabilities between health states [38]. The software uses differential equations based on incidence and mortality rates to calculate prevalence and case fatality rates for the diseases has been used in Global Burden of Disease (GBD) studies [39]. Epidemiological inputs, namely total population prevalent years lived with disability, incidence, prevalence and mortality associated with the included diseases were taken from GBD 2019 [40]. Morbidity impacts were quantified using prevalent years lived with disability multiplied by disease-specific disability weights from the GBD study [33].

Data on healthcare costs for either incident or prevalent cases were taken from the Australian Institute of Health and Welfare (AIHW). Costs for colon cancer and endometrial cancer (mean cost per incidence case) reported in 2001 prices [41] were inflated to 2019 prices using the Health Price Index [42, 43]. Healthcare costs of breast cancer, diabetes and stroke (mean cost per prevalent case) were calculated by dividing the total disease expenditure for each disease in 2015–2016 by the prevalence of that disease in 2015, by age and sex, as estimated by AIHW for the Australian Burden of Disease Study 2015 [44]. These costs were then inflated to 2019 values (Appendix 2) [43].

### Health impacts modelling

The impact of reducing population-level sitting time on SB-related chronic diseases was modelled to estimate the health gains and healthcare cost saving which would accrue over a one-year time horizon in the Australian adult population aged 18 years and above.

Modelling was undertaken in Excel 2013 and second order (parameter) uncertainty analyses were undertaken by applying Monte-Carlo simulations using the Excel add-in software, Ersatz version 1.35 (EpiGear International 2016). Two thousand iterations of the model were run and all results were presented with 95% uncertainty intervals (UI).

Four scenarios were tested. In scenario 1, we tested the health benefits gained and healthcare cost savings if 100% of Australian adults in the high SB group (> 8 h/day) and moderate group (4–8 h/d) reduced their sitting time to no more than 4 h per day. Scenario 2 and 3 tested the potential benefits that would result if 30% of persons in the high SB category shifted to the moderate category and 30% of people in the moderate category shifted to the low category. Under scenario 4, the effectiveness of the reduction in sitting time from a randomised controlled trial Stand Up Victoria [45] was modelled. Stand Up Victoria was a 12-month intensive multi-component SB intervention that included behavioural components (individual counselling and reinforcement follow up phone calls) and environmental changes (sit-stand workstation) [45]. Details of the intervention outcomes, feasibility and scalability can be found in previous publications by Healy et al. 2016 [42] and Gao et al. 2018 [16]. Under this scenario, threshold analysis was conducted to estimate the numbers of persons in both the moderate and high SB groups required to reduce their sitting time by 36.3 min to achieve same health benefits as in scenarios 2 and 3. Details of the scenarios are provided in Table 1 below. For all scenarios it was assumed the change in SB was maintained over the one year time horizon.

**Table 1:** Description of scenario analyses

High SB group	Moderate SB group
**Scenario 1**: 100% of both high SB group and moderate SB group reduce daily sitting time to < 4 h per day; i.e. average reduction of 6 h of sitting per day in high SB group; and average reduction of 2.8 h per day in moderate SB group	
**Scenario 2**: 30% of high SB group moved to moderate SB, i.e. average reduction of 1.4 h per day	**Scenario 3**: 30% of moderate SB group moved to low SB group, i.e. average reduction of 0.85 h per day
**Scenario 4**: A threshold analysis to estimate the numbers of persons in both the moderate and high SB groups required to reduce their sitting time by 36.3 min to achieve same health benefits as in scenarios 2 and 3	
SB: sedentary behaviour; High SB: sitting > 8 h daily; Moderate SB group: sitting between 4 – 8 h sitting daily; Low SB group: sitting < 4 h daily	

## Results

### Population exposure to sedentary behaviour

The analysis of the Australian NHS 2014–2015 [31] indicated that, on average, 36% of the Australian population (18 years and above) had moderate levels of sitting time (4–8 h per day), while 34% were sitting > 8 h. Overall, the mean sitting time was approximately 10 h per day within the high SB group and 5.8 h per day within the moderate SB group. Excessive sitting (> 8 h) was more prevalent in males (37%) than females (29%) as was moderate SB (39% and 35% respectively). Details of population sitting time (including both workplace and leisure sitting time) by sex, age group and by level of sedentary time are reported in Table 2 below.

**Table 2:** Mean self-reported sitting time for each category of sedentary behaviour in Australian adults, by sex and age

**Age** **(years)**	**Low SB—less than 4 h per day**			**Moderate SB—between 4 to 8 h per day**			**High SB – more than 8 h per day**	
	Average sitting time minutes per day Mean (SE)	Proportion Mean (SE)	Average sitting time minutes per day Mean (SE)		Proportion Mean (SE)	Average sitting time minutes per day Mean (SE)		Proportion SE proportion
Male								
18–24	171.27 (6.76)	27.47% (2.21%)	343.49 (5.12)		38.20% (2.39%)	685.28 (22.31)		34.33% (2.51%)
25–34	180.95 (4.48)	24.33% (1.84%)	342.76 (3.93)		31.71% (1.93%)	646.71 (9.63)		43.96% (1.89%)
35–44	180.06 (3.41)	24.81% (1.52%)	351.88 (3.57)		30.90% (2.06%)	634.14 (8.33)		44.30% (1.87%)
45–54	180.10 (4.21)	18.71% (1.46%)	354.70 (4.23)		35.06% (1.91%)	630.06 (7.79)		46.23% (2.07%)
55–64	194.87 (4.21)	25.72% (1.58%)	350.70 (3.74)		37.15% (1.93%)	653.30 (12.54)		37.13% (2.14%)
65–74	194.87 (3.44)	29.79% (1.69%)	354.59 (3.40)		42.31% (1.98%)	634.68 (16.07)		27.89% (1.90%)
75 +	186.70 (6.91)	26.02% (2.54%)	352.92 (3.58)		42.58% (2.71%)	623.90 (20.38)		31.40% (2.81%)
Female								
18–24	175.27 (4.50)	30.74% (2.51%)	362.73 (4.42)		35.01% (2.64%)	617.30 (12.28)		34.25% (2.69%)
25–34	164.47 (3.24)	34.92% (1.58%)	349.08 (3.61)		30.34% (1.58%)	621.41 (8.65)		34.73% (1.79%)
35–44	160.16 (3.06)	48.46% (1.41%)	348.97 (3.54)		25.06% (1.58%)	620.21 (7.76)		26.48% (1.27%)
45–54	163.02 (3.89)	38.50% (2.07%)	343.66 (2.89)		30.81% (1.76%)	618.50 (7.76)		30.69% (1.76%)
55–64	182.06 (3.54)	34.57% (1.57%)	340.54 (3.02)		39.16% (1.69%)	639.73 (9.81)		26.27% (1.48%)
65–74	173.92 (4.76)	33.30% (1.81%)	346.12 (2.59)		44.17% (1.96%)	627.60 (16.60)		22.53% (1.64%)
75 +	193.21 (4.12)	26.22% (2.12%)	356.87 (3.99)		43.80% (2.60%)	613.31 (9.88)		29.98% (2.20%)

*SB* sedentary behaviour, *SE* standard error

National Health Survey 2014–15 [31]

### Relative risk of SB-related diseases

A literature search identified five diseases with significantly increased risk associated with excessive sitting: breast cancer [46–49], colorectal cancer [50–52], endometrial cancer [53–55], T2D [56–61], stroke [62]. Each of the relative risk estimates from these studies were adjusted for individual physical activity levels and therefore represents the independent effect of SB on disease incidence. The results of the meta-analysis that provided a pooled estimate of the RRs of these diseases associated with SB categories are reported in Table 3. Details of the literature review and meta-analysis results are provided in in Appendix 1.

**Table 3:** The association between sitting time and chronic diseases

Disease	Number of studies	Meta-analysis results relative risk (95% CI)	
		**Moderate SB** **4–8 h sitting per day**	**High SB** ** > 8 h sitting per day**
**Results from meta-analyses – used as model inputs to calculate disease incidence**			
** Breast cancer**	4 cohort studies [50–52]	1.05 (0.94; 1.18)	1.22 (1.08; 1.39)
** Colorectal cancer**	3 cohort studies [50–52]	1.03 (0.94; 1.13)	1.16 (1.07; 1.26)
** Endometrial cancer**	3 cohort studies [50–52]	1.29 (0.99; 1.67)	1.54 (1.29; 1.83)
** T2D**	6 cohort studies [50–52]	1.13 (0.94; 1.35)	1.31 (1.15; 1.48)
**Results from a single cohort study**			
** Stroke**	1 cohort study [62]	1.05 (0.95; 1.15)	1.21 (1.07; 1.37)

*CI * confidence interval, *SB *sedentary behaviour,  *T2D *type 2 diabetes,  Reference group: Low sedentary behaviour, i.e. < 4 h of sitting per day

The association between sitting time and the risk of disease was not significant for moderate SB for all five diseases. However, the results of both moderate and high SB categories combined showed significant association (Appendix 1). Therefore, these non-significant RRs were included as model inputs.

### Potential health benefits and healthcare cost savings

The SB model inputs related to population size, all-causes mortality and healthcare costs are presented in Appendix 2. The modelling revealed that if all excessive SB was eliminated, i.e. people sitting < 4 h per day), a total of 17 211 (95%UI 14,758 to 19,923) HALYs and A$185 million (95%UI $164 M to $205 M) in healthcare cost could be saved over a one-year period. Under this scenario, a total of 3,204 deaths would be avoided, 27% of them from colorectal cancer, 26% from stroke, 22% from T2D, 17% from breast cancer and 8% from endometrial cancer. A total of 11 380 incidence cases of disease would be prevented, made up of T2D (58%), stroke (14%), colorectal cancer (13%), breast cancer (11%) and endometrial cancer (3%). Total incident cases of disease and deaths prevented were 5 864 and 1 858 in females versus 5 515 and 1 346 in males. However, there were more incident cases prevented and deaths avoided in males for T2D (3 824 deaths in males versus 2 799 in females) and colorectal cancer (878 deaths in males versus 564 females). Details of incident cases prevented by each disease for scenario 1 are reported in Appendix 3.

Under scenarios 2 and 3, where only 30% of the population shifted their SB level to the next lower category, total HALYs gained in the high SB and moderate SB group were 2 255 and 1 587 respectively. Associated healthcare cost savings were estimated to be A$24 million in the high SB group and A$17 million in the moderate SB group. Scenario 4 modelled the potential health benefits if people in both high and moderate SB groups (combined) could reduce their sitting time by 36.3 min per day [45]. HALYs gained and healthcare cost saving by sex are reported in Table 4 below.

**Table 4:** Health-adjusted life years gained and healthcare cost saving from reduction of population sitting time

	**Female** **Mean (95% UI)**	**Male** **Mean (95% UI)**		**Total** **Mean (95% UI)**	
	**Scenario 1:** 100% of adult population sat no more than 4 h daily				
HALYs gained	9 695 (8 282 to 11 127)		7 517 (6 279 to 8 966)		17 211 (14 758 to 19 923)
Health care cost-saving	$110 M ($96 M to $123 M)		$75 M ($65 M to $85 M)		$185 M ($164 M to $205 M)
	**Scenario 2:** 30% of persons in high SB group shifted to moderate SB				
HALYs gained	1 225 (1 058 to 1 412)		1 030 (854 to 1 217)		2 255 (1 933 to 2 606)
Health care cost-offset	$14 M ($12 M to $16 M)		$10 M ($9 M to $12 M)		$24 M ($21 M to $27 M)
	**Scenario 3:** 30% of persons in moderate SB group shifted to low SB				
HALYs gained	935 (801 to 1 077)		642 (527 to 771)		1 578 (1 342 to 1 835)
Health care cost-offset	$11 M ($9 M to $12 M)		$6 M ($6 M to $7 M)		$17 M ($15 M to $19 M)
	**Scenario 4:** A reduction in sitting time of 36.3 min daily across both high and moderate SB groups				
HALYs gained	2 213 (1 692 to 2 833)		1 457 (1 078 to 1 898)		3 670 (2 796 to 4 701)
Health care cost-offset	$25 M ($19 M to $32 M)		$15 M ($18 M to $11 M)		$39 M ($30 M to $50 M)

*HALY * health-adjusted life year,  *M *million,  *UI *uncertainty interval,  $: Australian dollar 2019

*SB *sedentary behaviour, High SB: sitting > 8 h daily; Moderate SB group: sitting between 4 – 8 h sitting daily; Low SB group: sitting < 4 h daily

## Discussion

Our study details the development of a SB model which can be widely used to estimate the potential health benefits and healthcare cost savings associated with reductions in SB in Australia. The model showed that excessive sitting time could cost the Australian healthcare system approximately A$185 million in 2019. Furthermore, it showed that 3 204 deaths could be prevented over one year if 100% of excessive SB was eliminated. Among the five SB-related diseases, T2D contributed to the most incident cases of disease prevented, followed by stroke and colorectal cancer. It is interesting that whilst SB is more prevalent in males, the HALYs gained are greater in females. This is likely because breast cancer and endometrial cancer in females are two of the five diseases included in the SB model.

While the above analyses inform the maximum burden of SB for Australia’s healthcare system in 2019, it is unrealistic to expect that interventions can fully eliminate excessive sedentary time. A more feasible expectation would be to have 30% of the high SB group reduce their sitting time to moderate levels (e.g. average reduction of 1.5 h per day in scenario 2) and 30% of moderate SB group moved to low SB levels (e.g. average reduction of 51 min in scenario 3). Under these two scenarios, substantial health grains and cost savings were predicted. A workplace intervention in Australia (Stand-Up-Victoria) reported a reduction in sitting time of 36.3 min per 16-h day [45]. When this effectiveness was modelled for both the high and moderate SB groups (scenario 4), the intervention would need to reach 60% of the adult population in order to achieve identical health benefits as in scenarios 2 and 3. The most recent systematic review with meta-analysis by Peachey et al. [13] reported a similar effect size (− 35.53 min/day; 95% CI − 57.27; –13.79) resulting from multi-component SB interventions including interventions outside of office settings (i.e. home or community settings). Thus, these effectiveness estimates require caution in interpretation given most of the trials included in the meta-analyses were rated as low quality or high risk of bias [11, 13, 14]. It is noteworthy that the low quality rating were mostly driven by the nature of office setting where blinding was not feasible [13, 14]. In relation to effectiveness, preventive interventions should also target people outside of an office setting.

Prior to our study, only one study has explored the magnitude of the health and economic burden of SB. Heron et al. 2019 [20] investigated the burden of excessive sitting to the UK health system in 2016–2017 and reported a cost-of-illness of GBP761.80 million. The UK study used a potential attributable fraction (PAF) approach and defined SB as sitting > 6 h per day [20], while we used a Monte Carlo simulation model with PIF to calculate the impact on disease incidence. Moreover, while Heron et al. [20] used a common RR for all CVDs combined, in our analysis only stroke was included in the model. Substantial differences in estimates between the two studies are not surprising given the population size differences (UK population 2016 versus Australia population 2019).

Our study has made several significant contributions to the academic literature. Previous modelling studies have estimated the health benefit of changes in SB through changes in physical activity, despite it being commonly accepted that SB and physical inactivity are not synonymous. Our study contributes new knowledge to the epidemiological and economic modelling by more accurately reflecting the changes in health outcomes that may directly result from any changes in SB levels. Secondly, our model is able to distinguish the benefits gained from different levels of time spent being sedentary, from high (> 8 h of sitting per day) to moderate level (4–8 h). Potentially, there are more opportunities to reduce sitting time in the high SB group who are likely to be sedentary during the day in workplaces, rather than those who already are sitting for shorter periods of time.

Our study also provides disease-level details on costs and RRs for SB-related diseases. The majority of research evidence on RRs is not at a disease-level, but instead uses one risk ratio for all CVDs, or for all cancers. Moreover, when assessing the association of excessive SB with diseases, we included only cohort studies reporting sitting time. We excluded cohort studies that explicitly investigated the risk of TV watching as several studies have shown TV watching to be associated with other adverse behaviours, such as excessive snack food intake [63]; and due to the differences between active screen time versus passive screen time [64]. For instance, the association between sitting time and T2D for those who are highly sedentary was estimated with a RR of 1.31 (95%CI 1.15; 1.48) in our study, but a HR of 1.91 (95%CI, 1.64 to 2.22) [3] was estimated when TV watching studies were included. Given the complexity of understanding any unique features of screen time that may impact the relationship between SB and chronic disease risk, we took a conservative approach by excluding screen time studies. Another strength of our study is that the RRs extracted for our meta-analyses were from a regression model where the risk of being sedentary was adjusted for physical activity levels. Our study also provides the most recent estimates of sitting time by Australian adults using data from the Australian NHS 2014 [31]. The high prevalence of excessive sitting time (more than 30% of adult’s population sit more than 8 h daily) highlights the need for policy action in this area.

This study is however not without limitations. The SB model is limited to diseases that were already incorporated in the ACE-Obesity Policy model [21, 22]. There are other potential SB-related diseases that were not included in this model, such as lung cancer [65], heart failure [66] and myocardial infarction [67]. Therefore our model is likely to underestimate the potential benefits associated with interventions that reduce sitting time. The model deals with health benefits from averting disease onset in the modelled cohort. The potential impact of reduced SB on disease progression is not captured in the health and cost savings estimates. This study shows that there are potentially substantial benefits of interventions to reduce sedentary behaviour – however the costs of these interventions need to be incorporated into the economic evaluation to demonstrate whether they represent good value for money.

Currently there is no consensus with respect to a specific cut-off for hours/day of SB at which the health risks associated with SB are most pronounced. Further research in this area is needed in order to specify a threshold that can be used consistently across studies and in line with SB guidelines. A well-defined cut-off will significantly impact the assessment of the association between SB and various diseases and help to guide future economic modelling. The most recent NHS in Australia 2019 [10] does not capture population sitting time as minutes per day or hour per week. Given SB is an established risk behaviour and distinct from physical inactivity [68], it is recommended that future Australian health surveys capture population sitting time in more detail.

## Conclusion

In conclusion, excessive sitting time contributes to considerable population health burden in Australia. This paper describes the development of the first Australian SB health and economic model that can be used to predict the long term consequences of interventions targeted at SB. This SB model can be used for cost-effectiveness analyses, which will be useful to decision makers when allocating resources to initiatives to reduce population time spent being sedentary. Future SB model development should include additional diseases and full economic evaluations of intervention studies which use more objective measures of sitting time.

## Supplementary Information

**Additional file 1:** **Appendix 1.** Literature review and meta-analyses of disease association

**Additional file 2:** **Appendix 2.** Model structure and inputs

**Additional file 3:** **Appendix 3.** Health impact modelling results

## Data Availability

All data generated or analysed during this study are included in this published article and its supplementary information files.

## Abbreviations

AIHW: Australian Institute of Health and Welfare
BMI: Body max index
CI: Confidence intervals
CURF: Confidentialised Unit Record Files
CVD: Cardiovascular disease
GBD: Global Burden of Disease
HALY: Health adjusted life year
HR: Hazard ratio
MET: Metabolic equivalent of task
PA: Physical activity
PIF: Potential impact fractions
RCT: Randomised controlled trial
REML: Restricted maximum likelihood method
SB: Sedentary behaviour
T2D: Type 2 diabetes
UI: Uncertainty interval
