# Supplementary material for: Modelling the potential health and economic benefits of reducing population sitting time in Australia
Source: Int J Behav Nutr Phys Act. 2022 Mar 19;19:28. doi: 10.1186/s12966-022-01276-2 (PMC8934131; doi:10.1186/s12966-022-01276-2)
Supplement: Supplementary file 1 — Additional file 1: Appendix 1. Literature review and meta-analyses of disease association [file 12966_2022_1276_MOESM1_ESM.docx]

# Appendix 1. Literature review and meta-analyses of disease association

## Objectives

In this current literature review with meta-analysis, we aimed to:

- Quantitatively synthesise cohort studies looking at the association of sedentary behaviour (SB) with different types of cancers, cardiovascular diseases (CVDs), type 2 diabetes (T2D) and other diseases to identify which diseases that have a significant association;
- Conduct a meta-analysis for each of the selected diseases (that included in the base ACE-Obesity Policy model) with three categories of SB level.

## Method

### Literature search and selection of reviews and cohort studies

Inclusion criteria

- Study design: cohort studies that report disease incidence (as a relative risk (RR), odd ratio (OR) or hazard ratio (HR))
- Outcome of interest: change in sitting time that covered all domains (work, home, leisure time and transport).
- Reported sitting time categories that can be fitted under our cut-off for SB level, i.e. less than 4 hours per day, between 4 to 8 hours, and more than 8 hours per day. For instance, if the highest level of SB reported in the original study was 3 hrs (1 hr less than our cut-off for the reference group), this study was excluded.

Exclusion criteria

- Studies that have case-control design or include people with conditions (stroke survivor, people with disabilities etc.). Studies that reported Odds Ratios, but did not provide summary data for RR calculation.
- Studies that did not report RRs adjusted for physical activity (PA) time

### Data extraction

To conduct our meta-analyses, we extracted Relative Risks (RR) and 95% Confidence Intervals for each level of sitting time from each study. It is important to note that the term “Relative Risk” may also be referred to as “Risk Ratio” which also leads to much confusion in literature. In order to conduct our meta-analyses, a number of assumptions were made about what was deemed appropriate to be referred to as a “Relative Risk (Risk Ratio)”. Although, there are technical differences in Hazard Rate/Ratios, Incidence Rate Ratios and Relative Risks ([1](#_ENREF_1)), we have assumed here in these analyses that they are appropriately similar. The majority of studies ([2-15](#_ENREF_2)) that met our inclusion criteria used a multivariable Cox Proportional Hazard regression model to produce an adjusted hazard ratio and 95% confidence Interval. In these studies, they have used the term “Relative Risk” to mean the same as a “Hazard Ratio”. Further, in studies ([16](#_ENREF_16), [17](#_ENREF_17)), the authors have conducted multivariable Poisson regressions and have reported “Incidence Risk Ratios” or “Incidence Rate Ratios”. Other included studies in the meta-analyses, also conducted multivariable Cox Proportional Hazard regression models but reported “Hazard Ratios”, while other two ([4](#_ENREF_4), [6](#_ENREF_6)) reported “Relative Risk” from their analyses. A subgroup sensitivity analysis was conducted (not included here but available upon request) to examine potential differences in effect sizes (Relative Risk). There were no studies that reported “Odds Ratios”, which potentially could have been converted to a “Relative Risk”. It was decided to proceed with meta-analysis. However, we highlight this in the discussion as a limitation of this meta-analysis.

The relative risk was chosen from the Cox Proportional Hazards and Poisson regression models which adjusted for PA. If there were options between body mass index (BMI) or non-BMI adjusted models, the result from the model that adjusted for PA, but not BMI, was chosen. Where sitting time was reported in hours per week, the result was divided by 7 to provide sitting time in hours per day. When allocating to categories, an interval of +/- 1 hrs range was accepted, i.e. 3-6 hrs cut-off was fitted in our moderate SB level 4-8 hrs. ORs were converted to RRs where possible.

### Statistical analysis

Separate meta-analyses were conducted for each outcome (breast cancer, colorectal cancer, endometrial cancers and Type 2 Diabetes) using Stata 16 ([18](#_ENREF_18)) and following the Prisma statement ([19](#_ENREF_19)). For all meta-analyses, Stata’s setup feature for precomputed effect size (logRR) and standard errors (logRRse) was used. Random-effect models using the Restricted Maximum Likelihood method (REML) was applied, pooling the effect size (ES) of included studies and reported Overall Relative Risk. Initially, all effect sizes extracted were converted to a logRR and a logRRse then back-transformed in the modelling for reporting purposes. The overall effect size was also back transformed to a Relative Risk and 95% Confidence Interval. Effects sizes used in the analyses compared the moderate level and high level of SB with low SB (as reference). Heterogeneity was assessed using Cochrane’s Q statistic (significance level set at 10%) and the I^2^ statistic based on Higgins and colleagues where 25%, 50%, and 75% represented low, moderate, and high heterogeneity, respectively. Given the scope of our research, publication bias was not assessed. A subgroup analysis for each outcome was conducted by high and moderate sedentary behaviour.

The literature search, data extraction and meta-analyses were conducted by PN.

## Results

**Literature search**

The literature search yielded a total number of 83 cohort studies; of these 19 were eligible for inclusion and 16 studies were included in the quantitative analysis. The Prisma flowchart is shown in Figure 2.1.

Meta-analysis were conducted for each disease where there was more than one cohort study. Among the included cohort studies, four studies investigated the association between sitting time and breast cancer ([3](#_ENREF_3), [5](#_ENREF_5), [15](#_ENREF_15), [20](#_ENREF_20)), three with colorectal cancer ([4](#_ENREF_4), [12](#_ENREF_12), [13](#_ENREF_13)), three with endometrial cancer ([2](#_ENREF_2), [6](#_ENREF_6), [11](#_ENREF_11)), six with T2D ([7-10](#_ENREF_7), [16](#_ENREF_16), [17](#_ENREF_17)) and one study for each disease: heart failure ([21](#_ENREF_21)), myocardial infraction ([22](#_ENREF_22)) and stroke ([23](#_ENREF_23)).

**Figure 1.1. PRISMA flowchart**


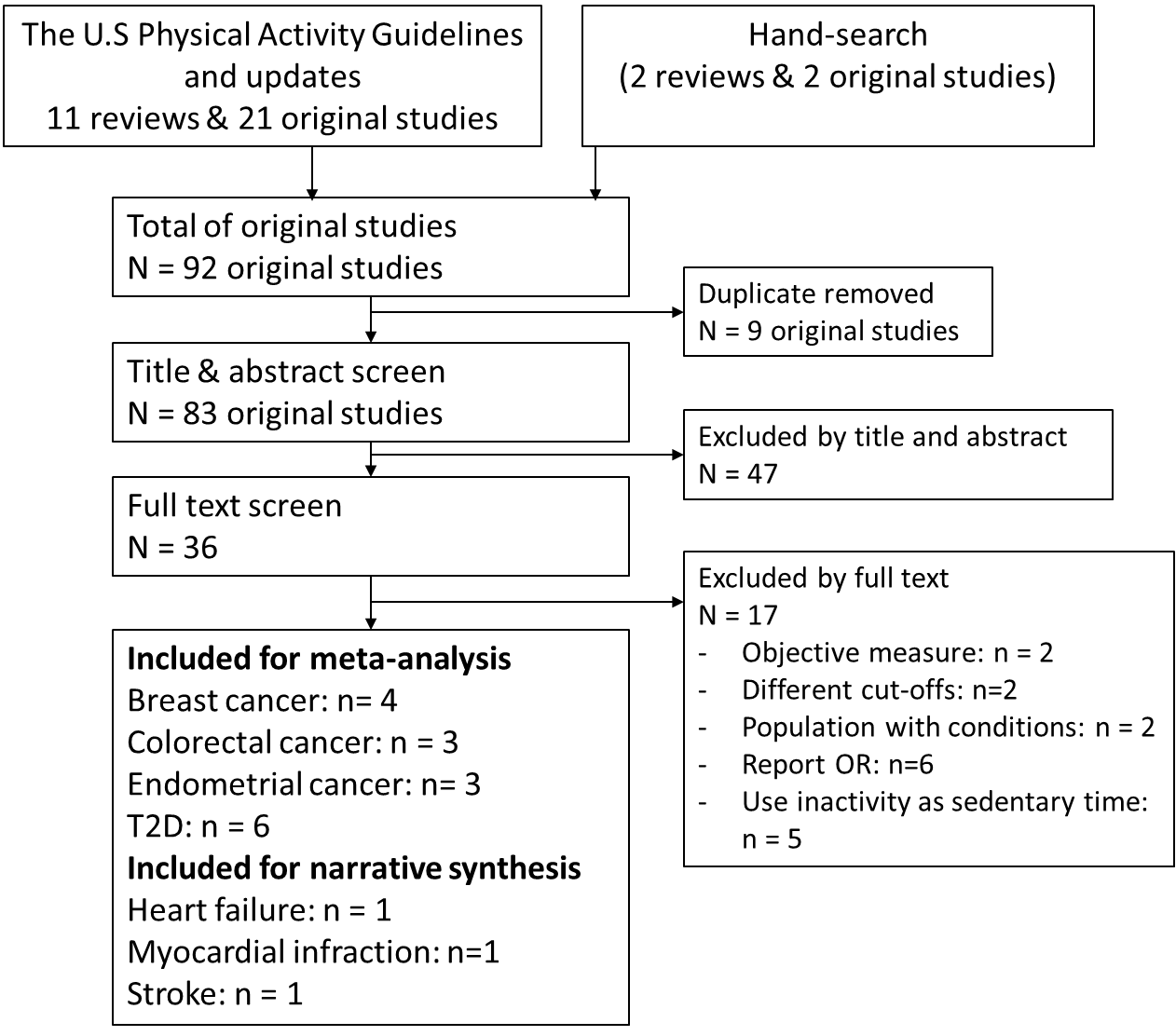


**Meta-analysis results**

***Breast cancer***

Figure 1.2. The association between sitting time and breast cancer


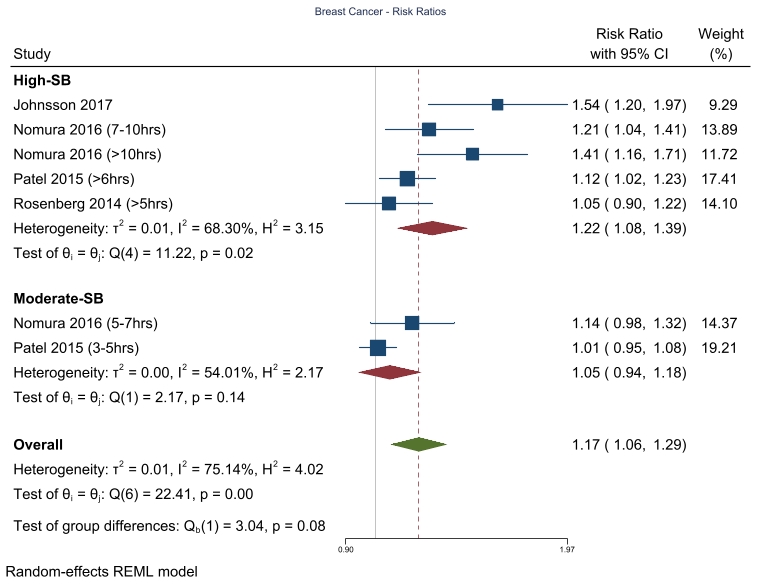


***Colorectal cancer***

Figure 1.3. The association between sitting time and colorectal cancer


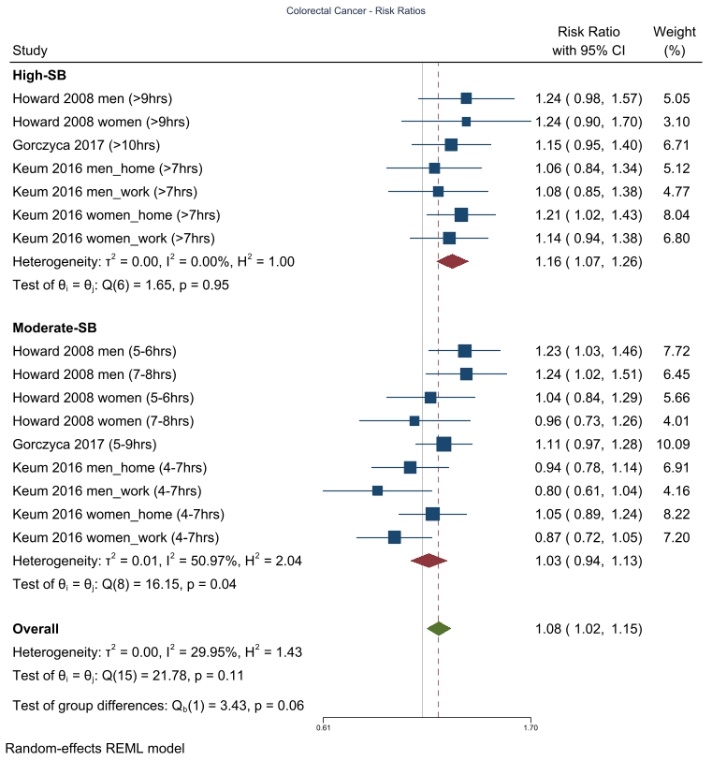


***Endometrial cancer***

Our literature search did not identify any new study other than what had been included in a previous meta-analysis conducted by Shen 2014. However, the meta-analysis by Shen et al reported only the highest level of SB, and the ES was chosen from a different regression model (adjusted for BMI). Therefore, an updated meta-analyses was conducted.

Figure 1.4. The association between sitting time and endometrial cancer


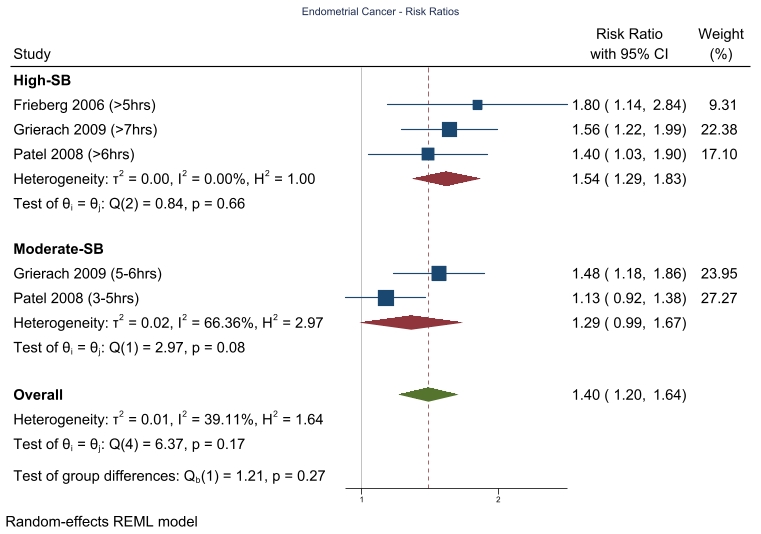


***Type 2 diabetes***

Our literature search identified two studies that reported OR convertible to RR; however inclusion of these two studies increased heterogeneity from 64% to 98%, so they were excluded from the final quantitative analysis.

Figure 1.5. The association between sitting time and diabetes


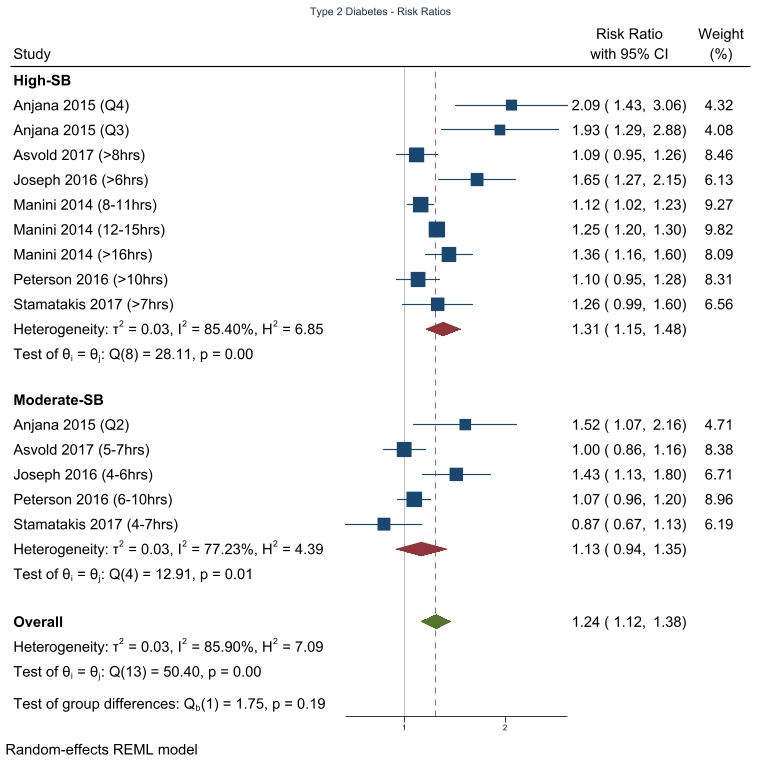


# Reference

1. Higgins JPT TJ, Chandler J, Cumpston M, Li T, Page MJ, Welch VA. Cochrane Handbook for Systematic Reviews of Interventions version 6.2 (updated February 2021)2021.

2. Patel AV, Feigelson HS, Talbot JT, McCullough ML, Rodriguez C, Patel RC, et al. The role of body weight in the relationship between physical activity and endometrial cancer: results from a large cohort of US women. International journal of cancer. 2008;123(8):1877-82.

3. Patel AV, Hildebrand JS, Campbell PT, Teras LR, Craft LL, McCullough ML, et al. Leisure-Time Spent Sitting and Site-Specific Cancer Incidence in a Large U.S. Cohort. Cancer Epidemiology Biomarkers &amp; Prevention. 2015;24(9):1350-9.

4. Howard RA, Freedman DM, Park Y, Hollenbeck A, Schatzkin A, Leitzmann MF. Physical activity, sedentary behavior, and the risk of colon and rectal cancer in the NIH-AARP Diet and Health Study. Cancer causes & control. 2008;19(9):939-53.

5. Rosenberg L, Palmer JR, Bethea TN, Ban Y, Kipping-Ruane K, Adams-Campbell LL. A Prospective Study of Physical Activity and Breast Cancer Incidence in African-American Women. Cancer Epidemiology Biomarkers &amp; Prevention. 2014;23(11):2522-31.

6. Friberg E, Mantzoros CS, Wolk A. Physical activity and risk of endometrial cancer: a population-based prospective cohort study. Cancer Epidemiology and Prevention Biomarkers. 2006;15(11):2136-40.

7. Åsvold BO, Midthjell K, Krokstad S, Rangul V, Bauman A. Prolonged sitting may increase diabetes risk in physically inactive individuals: an 11 year follow-up of the HUNT Study, Norway. Diabetologia. 2017;60(5):830-5.

8. Joseph JJ, Echouffo-Tcheugui JB, Golden SH, Chen H, Jenny NS, Carnethon MR, et al. Physical activity, sedentary behaviors and the incidence of type 2 diabetes mellitus: the Multi-Ethnic Study of Atherosclerosis (MESA). BMJ Open Diabetes Research &amp; Care. 2016;4(1):e000185.

9. Petersen CB, Bauman A, Tolstrup JS. Total sitting time and the risk of incident diabetes in Danish adults (the DANHES cohort) over 5 years: a prospective study. British journal of sports medicine. 2016;50(22):1382-7.

10. Stamatakis E, Pulsford RM, Brunner EJ, Britton AR, Bauman AE, Biddle SJ, et al. Sitting behaviour is not associated with incident diabetes over 13 years: the Whitehall II cohort study. British journal of sports medicine. 2017;51(10):818-23.

11. Gierach GL, Chang SC, Brinton LA, Lacey Jr JV, Hollenbeck AR, Schatzkin A, et al. Physical activity, sedentary behavior, and endometrial cancer risk in the NIH‐AARP Diet and Health Study. International journal of cancer. 2009;124(9):2139-47.

12. Gorczyca AM, Eaton CB, LaMonte MJ, Garcia DO, Johnston JD, He K, et al. Association of physical activity and sitting time with incident colorectal cancer in postmenopausal women. European journal of cancer prevention: the official journal of the European Cancer Prevention Organisation (ECP). 2018;27(4):331.

13. Keum N, Cao Y, Oh H, Smith‐Warner SA, Orav J, Wu K, et al. Sedentary behaviors and light‐intensity activities in relation to colorectal cancer risk. International journal of cancer. 2016;138(9):2109-17.

14. Nomura SJ, Dash C, Rosenberg L, Palmer J, Adams-Campbell LL. Sedentary time and breast cancer incidence in African American women. Cancer Causes & Control. 2016;27(10):1239-52.

15. Johnsson A, Broberg P, Johnsson A, Tornberg ÅB, Olsson H. Occupational sedentariness and breast cancer risk. Acta Oncologica. 2017;56(1):75-80.

16. Anjana RM, Sudha V, Nair DH, Lakshmipriya N, Deepa M, Pradeepa R, et al. Diabetes in Asian Indians—How much is preventable? Ten-year follow-up of the Chennai Urban Rural Epidemiology Study (CURES-142). Diabetes Research and Clinical Practice. 2015;109(2):253-61.

17. Manini TM, LaMonte MJ, Seguin RA, Manson JE, Hingle M, Garcia L, et al. Modifying effect of obesity on the association between sitting and incident diabetes in post‐menopausal women. Obesity. 2014;22(4):1133-41.

18. StataCorp'. Stata Statistical Software: Release 16. College Station. TX: StataCorp LLC2019.

19. Moher D, Altman DG, Liberati A, Tetzlaff J. PRISMA statement. Epidemiology. 2011;22(1):128.

20. Nomura SJO, Dash C, Sheppard VB, Bowen D, Allison M, Barrington W, et al. Sedentary time and postmenopausal breast cancer incidence. Cancer Causes & Control. 2017;28(12):1405-16.

21. Young DR, Reynolds K, Sidell M, Brar S, Ghai NR, Sternfeld B, et al. Effects of physical activity and sedentary time on the risk of heart failure. Circulation: Heart Failure. 2014;7(1):21-7.

22. Petersen CB, Bauman A, Grønbæk M, Helge JW, Thygesen LC, Tolstrup JS. Total sitting time and risk of myocardial infarction, coronary heart disease and all-cause mortality in a prospective cohort of Danish adults. International Journal of Behavioral Nutrition and Physical Activity. 2014;11(1):1-11.

23. Chomistek AK, Manson JE, Stefanick ML, Lu B, Sands-Lincoln M, Going SB, et al. Relationship of sedentary behavior and physical activity to incident cardiovascular disease: results from the Women's Health Initiative. Journal of the American College of Cardiology. 2013;61(23):2346-54.
