# Supplementary material for: Modelling the potential health and economic benefits of reducing population sitting time in Australia
Source: Int J Behav Nutr Phys Act. 2022 Mar 19;19:28. doi: 10.1186/s12966-022-01276-2 (PMC8934131; doi:10.1186/s12966-022-01276-2)
Supplement: Supplementary file 2 — Additional file 2: Appendix 2. Model structure and inputs [file 12966_2022_1276_MOESM2_ESM.docx]

# Appendix 2. Model structure and inputs

**Table 2.1: Australian 2019 Population, all-cause mortality rates by age and sex**

| Age | **Population 2019 (number)** | | **Mortality rate  (per 1,000)** | | **Age** | **Population 2019 (number)** | | **Mortality rate  (per 1,000)** | |
| --- | --- | --- | --- | --- | --- | --- | --- | --- | --- |
|  | **Male** | **Female** | **Male** | **Female** |  | **Male** | **Female** | **Male** | **Female** |
| 2 | 160,022 | 151,103 | 0.14 | 0.12 | 52 | 147,447 | 154,096 | 3.34 | 2.07 |
| 3 | 168,036 | 158,769 | 0.12 | 0.10 | 53 | 148,112 | 154,021 | 3.63 | 2.25 |
| 4 | 165,066 | 156,496 | 0.10 | 0.08 | 54 | 147,621 | 153,494 | 3.96 | 2.44 |
| 5 | 164,961 | 156,660 | 0.09 | 0.07 | 55 | 153,033 | 159,205 | 4.32 | 2.64 |
| 6 | 167,754 | 158,966 | 0.08 | 0.06 | 56 | 154,346 | 160,185 | 4.72 | 2.85 |
| 7 | 166,022 | 157,547 | 0.07 | 0.06 | 57 | 153,099 | 158,747 | 5.15 | 3.08 |
| 8 | 165,500 | 157,091 | 0.07 | 0.06 | 58 | 151,476 | 158,157 | 5.62 | 3.36 |
| 9 | 166,030 | 158,031 | 0.07 | 0.06 | 59 | 145,808 | 153,495 | 6.14 | 3.64 |
| 10 | 164,238 | 155,454 | 0.07 | 0.06 | 60 | 142,162 | 149,055 | 6.69 | 3.97 |
| 11 | 164,001 | 155,980 | 0.08 | 0.07 | 61 | 139,089 | 147,074 | 7.28 | 4.32 |
| 12 | 162,685 | 153,892 | 0.10 | 0.09 | 62 | 135,277 | 142,668 | 7.91 | 4.66 |
| 13 | 156,879 | 148,572 | 0.13 | 0.11 | 63 | 133,444 | 140,064 | 8.56 | 4.99 |
| 14 | 151,355 | 142,780 | 0.17 | 0.13 | 64 | 127,085 | 135,386 | 9.25 | 5.36 |
| 15 | 149,777 | 140,919 | 0.24 | 0.16 | 65 | 123,209 | 132,197 | 10.00 | 5.83 |
| 16 | 147,801 | 140,849 | 0.31 | 0.18 | 66 | 122,149 | 129,701 | 10.83 | 6.39 |
| 17 | 148,642 | 141,434 | 0.40 | 0.20 | 67 | 118,843 | 125,122 | 11.76 | 7.05 |
| 18 | 157,879 | 148,710 | 0.48 | 0.22 | 68 | 117,572 | 123,550 | 12.81 | 7.79 |
| 19 | 166,718 | 156,753 | 0.54 | 0.23 | 69 | 114,485 | 120,269 | 14.01 | 8.60 |
| 20 | 169,971 | 158,969 | 0.58 | 0.24 | 70 | 110,931 | 115,431 | 15.39 | 9.54 |
| 21 | 171,441 | 162,348 | 0.60 | 0.24 | 71 | 111,519 | 115,074 | 16.95 | 10.61 |
| 22 | 176,744 | 168,691 | 0.62 | 0.24 | 72 | 113,431 | 117,274 | 18.70 | 11.86 |
| 23 | 186,402 | 176,770 | 0.63 | 0.24 | 73 | 94,700 | 98,305 | 20.68 | 13.26 |
| 24 | 194,745 | 184,195 | 0.64 | 0.24 | 74 | 88,247 | 93,118 | 22.89 | 14.81 |
| 25 | 192,992 | 185,192 | 0.65 | 0.24 | 75 | 82,801 | 88,158 | 25.42 | 16.58 |
| 26 | 190,426 | 185,974 | 0.66 | 0.26 | 76 | 73,018 | 78,977 | 28.35 | 18.56 |
| 27 | 189,667 | 188,638 | 0.67 | 0.27 | 77 | 71,224 | 76,981 | 31.75 | 20.81 |
| 28 | 191,483 | 193,056 | 0.69 | 0.28 | 78 | 64,276 | 71,403 | 35.68 | 23.46 |
| 29 | 193,224 | 196,311 | 0.72 | 0.28 | 79 | 59,758 | 67,738 | 40.14 | 26.53 |
| 30 | 188,508 | 193,557 | 0.75 | 0.30 | 80 | 54,691 | 63,677 | 45.18 | 30.16 |
| 31 | 187,823 | 193,035 | 0.79 | 0.33 | 81 | 49,952 | 59,522 | 50.85 | 34.44 |
| 32 | 185,761 | 190,662 | 0.83 | 0.37 | 82 | 46,115 | 55,708 | 57.27 | 39.42 |
| 33 | 187,026 | 192,019 | 0.88 | 0.41 | 83 | 41,250 | 51,667 | 64.58 | 45.19 |
| 34 | 184,474 | 190,044 | 0.94 | 0.45 | 84 | 35,913 | 46,592 | 73.00 | 52.00 |
| 35 | 184,129 | 188,093 | 1.00 | 0.50 | 85 | 129,207 | 184,648 | 82.61 | 59.80 |
| 36 | 183,168 | 185,991 | 1.07 | 0.54 | 86 |  |  | 93.40 | 68.51 |
| 37 | 178,150 | 179,799 | 1.14 | 0.58 | 87 |  |  | 105.49 | 78.49 |
| 38 | 173,471 | 174,929 | 1.21 | 0.64 | 88 |  |  | 119.00 | 89.70 |
| 39 | 166,481 | 168,077 | 1.29 | 0.70 | 89 |  |  | 133.97 | 102.58 |
| 40 | 161,350 | 163,374 | 1.38 | 0.76 | 90 | 53,978 | 99,014 | 150.08 | 117.17 |
| 41 | 158,205 | 160,277 | 1.47 | 0.82 | 91 |  |  | 167.05 | 132.99 |
| 42 | 157,081 | 158,759 | 1.57 | 0.90 | 92 |  |  | 184.46 | 149.85 |
| 43 | 158,439 | 159,682 | 1.70 | 0.99 | 93 |  |  | 201.60 | 167.81 |
| 44 | 158,490 | 160,472 | 1.84 | 1.07 | 94 |  |  | 217.51 | 187.00 |
| 45 | 162,796 | 164,940 | 2.00 | 1.16 | 95 | 12,470 | 30,660 | 225.28 | 197.43 |
| 46 | 164,305 | 168,914 | 2.17 | 1.25 | 96 |  |  | 244.31 | 222.85 |
| 47 | 169,131 | 175,236 | 2.33 | 1.36 | 97 |  |  | 263.34 | 238.74 |
| 48 | 169,653 | 178,217 | 2.50 | 1.48 | 98 |  |  | 282.37 | 252.76 |
| 49 | 159,691 | 166,654 | 2.68 | 1.62 | 99 |  |  | 301.40 | 267.74 |
| 50 | 156,126 | 164,456 | 2.87 | 1.76 | 100+ | 1,406 | 3,586 | 320.43 | 283.85 |
| 51 | 151,396 | 158,783 | 3.09 | 1.91 |  |  |  |  |  |
| Source: ABS State and Territories Population 2019 ([1](#_ENREF_1)); ABS Lifetables 2019 ([2](#_ENREF_2)) | | | | | | | | | |

The prevalence and mean sitting time of each SB category were taken from the Australian National Health Survey 2014-2015 ([3](#_ENREF_3)) and reported in Appendix 1; the relative risk (RR) estimates were taken from meta-analyses reported in Appendix 2.

## **Disease models**

**Figure 2.2. Each disease is simulated with four health states (adapted from Barendregt et al 2003 (**[4](#_ENREF_4)**)).**

##

## **Disease costs**

Healthcare costs by disease, sex and age groups are reported in Table 3.2 below

**Table 2.2: Health care costs by disease**

| **sex** | **Age (years)** | **Colon Cancer** | **Breast Cancer** | **Endometrial Cancer** | **Stroke** | **Diabetes** | **Kidney cancer** | **Ischaemic heart disease** | **Hypertensive heart disease** | **Osteoarthritis** |
| --- | --- | --- | --- | --- | --- | --- | --- | --- | --- | --- |
| Male | 15-19 |  |  |  | $8 843 | $6 052 | $23 687 | $39 918 |  |  |
|  | 20-24 |  |  |  | $4 542 | $3 073 | $23 687 | $12 857 |  |  |
|  | 25-29 |  |  |  | $2 581 | $1 870 | $23 687 | $8 674 |  |  |
|  | 30-34 |  |  |  | $3 119 | $1 567 | $23 687 | $5 189 |  |  |
|  | 35-39 |  | $2 747 |  | $3 728 | $1 265 | $23 687 | $4 591 |  |  |
|  | 40-44 |  | $10 081 |  | $2 857 | $1 209 | $23 687 | $3 614 | $5 441 | $720 |
|  | 45-49 |  | $8 884 |  | $3 503 | $1 088 | $23 687 | $3 201 | $2 741 | $731 |
|  | 50-54 | $25 420 | $5 464 |  | $2 589 | $835 | $23 687 | $3 069 | $4 959 | $731 |
|  | 55–59 | $25 662 | $6 116 |  | $2 925 | $766 | $24 346 | $2 855 | $1 776 | $1 605 |
|  | 60-64 | $25 662 | $3 160 |  | $2 643 | $790 | $24 346 | $2 778 | $1 250 | $1 605 |
|  | 65–69 | $26 399 | $6 022 |  | $2 551 | $906 | $21 435 | $2 660 | $2 559 | $2 825 |
|  | 70-74 | $26 399 | $4 422 |  | $2 722 | $1 059 | $21 435 | $2 567 | $1 809 | $2 825 |
|  | 75- 79 | $26 215 | $5 149 |  | $2 909 | $1 282 | $21 113 | $2 361 | $936 | $3 024 |
|  | 80-84 | $26 215 | $5 383 |  | $3 037 | $2 063 | $21 113 | $1 966 | $1 306 | $3 024 |
|  | 85+ | $28 033 | $2 654 |  | $2 784 | $2 324 | $10 714 | $1 438 | $637 | $1 384 |
| Female | 15-19 |  |  |  | $11 084 | $8 737 | $22 534 | $35 594 |  |  |
|  | 20-24 |  | $205 773 |  | $6 391 | $5 833 | $22 534 | $25 209 |  |  |
|  | 25-29 |  | $27 354 |  | $3 750 | $4 074 | $22 534 | $18 819 |  |  |
|  | 30-34 |  | $14 637 |  | $4 341 | $3 132 | $22 534 | $14 287 |  |  |
|  | 35-39 |  | $11 890 |  | $3 736 | $2 066 | $22 534 | $7 786 |  |  |
|  | 40-44 |  | $9 812 |  | $3 704 | $1 145 | $22 534 | $5 090 | $2 424 | $350 |
|  | 45-49 |  | $7 283 |  | $3 967 | $976 | $22 534 | $3 960 | $3 463 | $498 |
|  | 50-54 | $24 906 | $6 223 | $15 500 | $3 391 | $970 | $22 534 | $3 209 | $3 808 | $498 |
|  | 55–59 | $23 762 | $5 919 | $14 391 | $3 644 | $856 | $23 781 | $2 488 | $1 606 | $1 222 |
|  | 60-64 | $23 762 | $5 420 | $14 391 | $3 368 | $877 | $23 781 | $2 394 | $1 951 | $1 222 |
|  | 65-69 | $25 054 | $4 710 | $20 956 | $3 108 | $890 | $24 901 | $2 337 | $2 625 | $2 215 |
|  | 70-74 | $25 054 | $4 471 | $20 956 | $3 188 | $914 | $24 901 | $2 172 | $1 602 | $2 215 |
|  | 75-79 | $25 231 | $3 521 | $15 256 | $3 038 | $1 075 | $24 996 | $2 009 | $2 300 | $2 466 |
|  | 80-84 | $25 231 | $3 291 | $15 256 | $3 013 | $1 632 | $24 996 | $1 635 | $1 439 | $2 466 |
|  | 85+ | $24 046 | $2 558 | $19 479 | $2 777 | $1 673 | $17 719 | $1 111 | $1 116 | $1 038 |

# Reference

1. Australian Bureau of Statistics. 31010DO002_202003 National, state and territory, Mar 2020. In: ABS, editor. Canberra2020.

2. Australian Bureau of Statistics. Lifetables. In: ABS, editor. 2017-2019. Canberra2020.

3. Australian Bureau of Statistics. 4324.0.55.001 - Microdata: National Health Survey, 2014-15. In: ABS, editor. Canberra2016.

4. Barendregt JJ, Van Oortmarssen GJ, Vos T, Murray CJ. A generic model for the assessment of disease epidemiology: the computational basis of DisMod II. Population health metrics. 2003;1(1):1-8.
