# Supplementary material for: Modelling the potential health and economic benefits of reducing population sitting time in Australia
Source: Int J Behav Nutr Phys Act. 2022 Mar 19;19:28. doi: 10.1186/s12966-022-01276-2 (PMC8934131; doi:10.1186/s12966-022-01276-2)
Supplement: Supplementary file 3 — Additional file 3: Appendix 3. Health impact modelling results [file 12966_2022_1276_MOESM3_ESM.docx]

# Appendix 3. Health impact modelling results

Table 3.1. Case prevented by diseases over one year

| **Case prevented**  Mean (95% uncertainty interval) | | **Scenario 1: Adult population sit no more than 4 hour per day** | | |
| --- | --- | --- | --- | --- |
|  |  | Female | Male | Total |
| Breast cancer | Incidence | 1,285 (963 to 1,598) |  | 1,285 (963 to 1,598) |
|  | Prevalence | 22,451 (16,294 to 28,426) |  | 22,451 (16,294 to 28,426) |
|  | Death | 547 (400 to 690) |  | 547 (400 to 690) |
| Colorectal cancer | Incidence | 564 (420 to 701) | 878 (673 to 1,077) | 1,443 (1,097 to 1,780) |
|  | Prevalence | 5,576 (4,308 to 6,784) | 8,170 (6,369 to 9,908) | 13,747 (10,666 to 16,675) |
|  | Death | 340 (250 to 428) | 511 (390 to 628) | 851 (644 to 1,054) |
| Endometrial cancer | Incidence | 386 (301 to 472) |  | 386 (301 to 472) |
|  | Prevalence | 4,908 (3,814 to 6,034) |  | 4,908 (3,814 to 6,034) |
|  | Death | 247 (190 to 304) |  | 247 (190 to 304) |
| Diabetes | Incidence | 2,799 (2,252 to 3,333) | 3,824 (3,067 to 4,554) | 6,623 (5,338 to 7,857) |
|  | Prevalence | 76,740 (62,277 to 90,486) | 100,698 (81,455 to 119,547) | 177,438 (143,719 to 209,956) |
|  | Death | 303 (237 to 368) | 417 (330 to 501) | 720 (572 to 865) |
| Stroke | Incidence | 830 (600 to 1,073) | 813 (604 to 1,025) | 1,644 (1,207 to 2,092) |
|  | Prevalence | 11,252 (8,656 to 13,878) | 9,840 (7,544 to 12,131) | 21,092 (16,141 to 25,929) |
|  | Death | 420 (282 to 564) | 418 (304 to 535) | 838 (590 to 1,097) |
